# Evidence of Common Isolates of *Streptococcus agalactiae* in Bovines and Humans in Emilia Romagna Region (Northern Italy)

**DOI:** 10.3389/fmicb.2021.673126

**Published:** 2021-06-11

**Authors:** Elena Carra, Simone Russo, Alessia Micheli, Chiara Garbarino, Matteo Ricchi, Federica Bergamini, Patrizia Bassi, Alice Prosperi, Silvia Piva, Monica Cricca, Roberta Schiavo, Giuseppe Merialdi, Andrea Serraino, Norma Arrigoni

**Affiliations:** ^1^Experimental Zooprophylactic Institute in Lombardy and Emilia Romagna, Brescia, Italy; ^2^Department of Veterinary Medical Sciences, University of Bologna, Bologna, Italy; ^3^Microbiology, DIMES, Alma Mater Studiorum, University of Bologna, Bologna, Italy; ^4^Center for Applied Biomedical Research, St. Orsola-Malpighi University Hospital, Bologna, Italy; ^5^Microbiology, Department of Clinical Pathology, “Guglielmo da Saliceto” Hospital, Piacenza, Italy

**Keywords:** *Streptococcus agalactiae*, bovines, humans, genotyping, MLST, pilus island, molecular capsular typing, antimicrobial resistance

## Abstract

*Streptococcus agalactiae* (group B *Streptococcus*, GBS) is one of the most important agents of bovine mastitis and causes remarkable direct and indirect economic losses to the livestock sector. Moreover, this species can cause severe human diseases in susceptible individuals. To investigate the zoonotic potential of *S. agalactiae*, 203 sympatric isolates from both humans and cattle, isolated in the same time frame (2018) and in the same geographic area (Emilia Romagna region, Northern Italy), were characterized by molecular capsular typing (MCT), pilus island typing (PI), and multi-locus sequence typing (MLST). In addition, antibiotic-resistant phenotypes were investigated. The distribution of the allelic profiles obtained by combining the three genotyping methods (MCT-PI-MLST) resulted in 64 possible genotypes, with greater genetic variability among the human compared to the bovine isolates. Although the combined methods had a high discriminatory power (>96,2%), five genotypes were observed in both species (20,9% of the total isolates). Furthermore, some of these strains shared the same antibiotic resistance profiles. The finding of human and bovine isolates with common genotypes and antibiotic resistance profiles supports the hypothesis of interspecies transmission of *S. agalactiae* between bovines and humans.

## Introduction

*Streptococcus agalactiae* (Group B Streptococcus, GBS) is one of the etiological agents of bovine contagious mastitis. The disease causes considerable direct and indirect economic losses to the livestock sector (Hogeveen et al., 2011; Åkerstedt et al., 2012). During the 1950s, this bacterium was the leading cause of mastitis in Europe (Katholm et al., 2012; Mweu et al., 2012). After the application of control plans from 1960–2000, the prevalence of the infection gradually decreased, leading to disease eradication in some countries (Skoff et al., 2009; Lambertsen et al., 2010; Katholm et al., 2012).

In the 21st century, due to major changes in dairy farm management in most European countries, such as reductions in the number of farms, increased herd sizes, introduction of robotic milking systems, and selective antibiotic treatments at drying off, the prevalence of *S*. *agalactiae* infection in cattle has increased, and is thus considered a re-emerging problem (Skoff et al., 2009; Lambertsen et al., 2010; Katholm et al., 2012).

In Italy, the infection is widespread in the bovine population, with an estimated herd-level prevalence of 7–10% in Lombardy and Emilia-Romagna (personal data).

*Streptococcus agalactiae* is a commensal species of human gastrointestinal and genitourinary flora and colonizes the gastrointestinal and genitourinary tracts of 10–35% of the adult human population. The asymptomatic colonization of the large intestine and genitourinary tract of pregnant women is the main cause of infection in new-borns during childbirth (Le Doare and Heath, 2013). Moreover, it can also cause bacteremia, skin and soft tissue infections, urinary tract infections, and occasionally necrotizing fasciitis, arthritis, toxic shock syndrome, endocarditis, meningitis, and pneumonia (Sendi et al., 2008, 2011; Sunkara et al., 2012).

In this context, epidemiological studies have excluded the transmission of *S. agalactiae* between humans and cattle (Bisharat et al., 2004; Richards et al., 2011; Bergal et al., 2015; Emaneini et al., 2016); however, a recent study (Lyhs et al., 2016) that analyzed a large number of sympatric field isolates from humans and cattle highlighted how the same subtypes were present in both species, bringing back the attention of the scientific community about possible interspecies transmission of GBS. In this regard, another recent study employing a phylogenetic approach confirmed that some GBS can be transmitted between cattle and humans (Botelho et al., 2018). The same authors hypothesized that transmission to humans can occur during milking, drinking contaminated milk, or through environmental contamination.

Several methods for identifying and characterizing GBS for diagnostic and epidemiological purposes have been described. These include molecular capsular typing (MCT), multi-locus sequence typing (MLST), typing of surface proteins (such as virulence factors), and typing of surface pili that mediate interactions with host cells (Shabayek and Spellerberg, 2018). Three pilus islands (PI), PI-1, PI-2a, and PI-2b, which encode distinct pilus structures that mediate interactions with host cells, have been identified on the GBS surface (Springman et al., 2014). The MLST profiling scheme is publicly available and regularly updated (Springman et al., 2014; Furfaro et al., 2018), allowing the worldwide comparison of GBS field isolates (Jones et al., 2003).

Our research aimed to investigate whether *S. agalactiae* isolates circulating in cattle and humans shared the same genotyping profiles and the same antibiotic-resistant phenotypes. For this purpose, sympatric field isolates from dairy cattle and human patients from hospitals located in the same territorial area (Emilia Romagna Region, Northern Italy) were compared using combined molecular subtyping methods.

## Materials and Methods

### Bovine and Human Isolates

From January to September 2018, during routine diagnostic activity at the Istituto Zooprofilattico Sperimentale della Lombardia and Emilia Romagna (IZSLER) Laboratories, 191 *S. agalactiae* isolates were collected from the milk of individual cows from 49 different herds from five provinces of the Emilia-Romagna region (Piacenza, Parma, Reggio Emilia, Modena, Bologna). Phenotypic identification was performed according to the recommendations of the National Mastitis Council (Adkins et al., 2017). At least three isolates were selected for each herd, choosing those showing different serotypes (for the definition see below), except for two herds located in the Parma province, in which four isolates were selected because they all belonged to different serotypes. All isolates came from the diagnostic activity of all lactating cows of infected herds submitted to eradication plans. The sampled animals were affected by subclinical infections and were only characterized by high somatic cell counts (SCC). This presentation of the disease is most common in cattle (Adkins et al., 2017). For the purposes of the eradication plans, the SCC data were not collected and were not relevant for subsequent actions (isolation, separate milking, or antimicrobial therapy). Based on this criterion, 103 isolates were selected for molecular typing: 53 isolates from 28 herds in Parma province, 20 isolates from nine herds in Reggio Emilia province, 15 isolates from seven herds in Piacenza province, 12 isolates from four herds in Modena province, and three isolates from one herd in Bologna province (Table 1).

**TABLE 1:** Origin and characteristics of *Streptococcus agalactiae* isolates included in this study.

Host	Number of isolates	Geographical origin (province)	Number of herds	Number of MCT-PI- MLST profiles
Bovine	53	Parma	28	18*
	20	Reggio Emilia	9	7
	15	Piacenza	7	8
	12	Modena	4	4
	3	Bologna	1	1
TOTAL	**103**		**49**	

**Host**	**Number of isolates**	**Geographical origin (province)**	**Number of hospitals**	**Number of MCT-PI- MLST profiles**

Humans	49	Piacenza	1	24*
	51	Bologna	1	27
TOTAL	**100**		**2**	

*With the exclusion of one isolate from an incomplete MLST profile. MCT, molecular capsular typing; PI, pilus island typing; MLST, multi-locus sequence typing. The numbers in bold represent the total of bovine and human Isolates in the second column, respectively, and the numbers of herds and hospitals in the fourth column.

In the same period (February–July 2018), 100 *S. agalactiae* isolates of human origin were collected. In detail, 49 patients were from the Guglielmo da Saliceto Hospital, in Piacenza province, and 51 from patients of the Polyclinic S. Orsola-Malpighi in Bologna province. These isolates were from different sources, mainly vaginal swabs and vaginal–rectal swabs (71,0%), collected in the context of surveillance of pregnant women, and from urine, urethral swabs, and other sources (29,0%) from symptomatic patients.

Human urines and genital swabs were processed as follow: one μL of urine was seeded in Horse Blood Agar media (Vacutest Kima, Italy), selective (Chrome Candida, Vacutest Kima, Italy) and non-selective chromogenic media (Chrome Orientation, Vacutest Kima, Italy) and incubated at 37°C for 24 and 48, respectively. Genital swabs were directly streaked in the above described media. Ano-genital swabs were collected for *S. agalactiae* screening during late pregnancy, enriched in Lim Broth (Copan, Italy) and incubated at 37°C for 16–24 h, then 10 μL of broth were seeded in chromogenic agar plate (ChromeAgar StrepB, Vacutest Kima, Italy or in ChromID^®^ Strepto B agar, Biomeriéux, Italia) and incubated at 37°C for 24 h.

*Streptococcus agalactiae* suspected colonies were sub-cultured onto tryptone soy agar media with sheep blood (Vacutest Kima, Italy) for 16 h of incubation, then the bacterial isolates were identified by MALDI-TOF Mass Spectrometry (Bruker, Milan, Italy). Finally, the clinical isolates were frozen at −70°C using MicroBank vials (Pro-Lab Diagnostics, Round Rock, TX, United States).

The details of the isolation matrices and the characteristics of all bovine and human isolates included in this study are listed in Supplementary Tables 1, 2, respectively.

### Species Identification and Lactose Typing

DNA was extracted from each isolate using a semi-automatic method. Briefly, a few colonies were suspended in 100 μL of distilled water and then processed according to the One-For-All-Vet kit (Qiagen, Milan, Italy).

To confirm the phenotypic identification of *S. agalactiae*, the extracted DNA was subjected to species-specific PCR (Lyhs et al., 2016).

Subsequently, all isolates were subjected to phenotypic lactose typing (LT), testing the ability of each GBS to metabolize lactose. For this purpose, some colonies of each isolate were suspended in phenol red broth (BioLife, Milan, Italy) supplemented with lactose monohydrate (Carlo Erba, Val de Reuil, France) and incubated for up to 7 days at 37°C (Lyhs et al., 2016).

### Molecular Capsular Typing

A multiplex MCT PCR assay was performed to detect all known GBS capsular polysaccharides (s) according to Poyart et al. (2007).

### Pilus Island Typing

**FIGURE 1:**
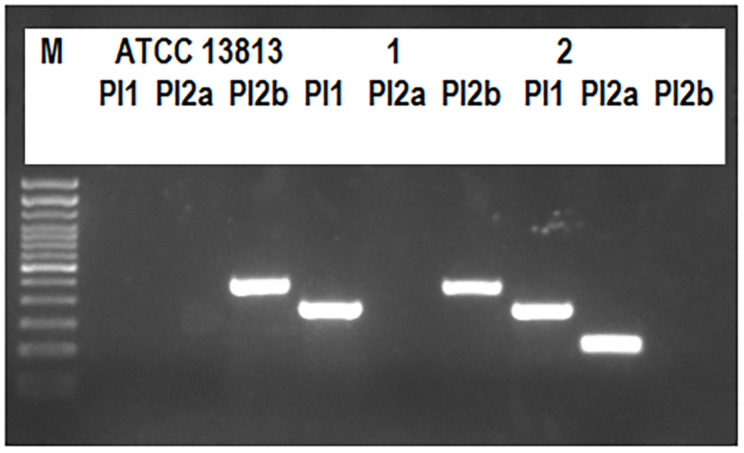
Pilus island typing. The expected fragment sizes were 394, 243, and 519 bp for PI-1, PI-2a, or PI-2b, respectively. The image shows the pilus island profiles obtained for the ATCC 13813 strain, and for the human 1–109 (1) and 2–109 (2) isolates.

Multiplex PCR was used to screen for the presence of PI genes in a final volume of 25 μL with three primer pairs (PI-1: SAG647_F496, SAG647_R889; PI 2a: SAG1406_F356, SAG1406_R598; PI 2b: SAN1517_F57, SAN1517_R575) as previously described (Springman et al., 2014), using the Mastermix GoTaq^®^ G2 Hot Start Colorless (Promega, Madison, WI). The amplification products were electrophoresed on a 1.8% agarose gel stained with Midori Green Advance (NIPPON Genetics Europe, Deuren, Germany) in 0.5× TBE running buffer (Sigma-Aldrich, St. Louis, MO, United States) at a constant voltage (90 V) in the presence of a 100-bp DNA ladder (Invitrogen, Carlsbad, CA, United States) and visualized on an ultraviolet trans-illuminator. The expected size fragments were 394, 243, and 519 bp for PI-1, PI-2a, and PI-2b, respectively (Figure 1). For PI-1, the presence of a 684-bp amplicon indicated an intact integration site; otherwise, the absence of an amplicon indicated occupation by an alternative, uncharacterized genetic element (Springman et al., 2014). The method was also applied to the ATCC 13813 *S. agalactiae* strain (Figure 1) as a positive control.

### Multi-Locus Sequence Typing

The MLST genotyping protocol of the selected *S. agalactiae* isolates was performed according to a previous study (Jones et al., 2003). In detail, seven housekeeping genes encoding alcohol dehydrogenase (adhP), phenylalanine tRNA synthetase (pheS), amino acid transporter (atr), glutamine synthetase (glnA), serine dehydratase (sdhA), glucose kinase (glcK), and transketolase (tkt) were genotyped. PCR reactions were performed in 10 μL volumes using the Mastermix GoTaq^®^ G2 Hot Start Colorless (Promega). The PCR conditions were 95°C for 2 min, followed by 30 cycles at 94°C for 1 min, 55°C for 45 s, and 72°C for 1 min, and 5 min at 72°C before cooling at 4°C. PCR products were purified by enzyme treatment with a mixture of exonuclease I and shrimp alkaline phosphatase (GE Healthcare, Little Chalfont, United Kingdom). Purified PCR products were sequenced in both directions using the GenomeLab DTCS Quick Start Kit (Beckman Coulter, Indianapolis, IN, United States) in a final volume of 10 μL using sequencing primers according to the above reference (Jones et al., 2003). Sequencing was performed with a Sciex GenomeLab^TM^ GeXP sequencer (Beckman Coulter), and the results were analyzed and assembled using the “Sequencing” and “Investigator” packages of GenomeLab^TM^ System software, version 11.0.24. Alleles and sequence type (ST) were assigned using the *S. agalactiae* database^1^ (Jolley et al., 2018). Isolates were assigned an ST according to their allelic profiles. New alleles were submitted to the database curator for quality control and the allocation of allele numbers and STs. Novel allele combinations were also submitted for ST assignment.

### Data Analysis

Global eBURST analysis was performed using PHYLOVIZ software (available online) (Francisco et al., 2012): isolates sharing the same alleles in at least five of seven loci were included in the same clonal complex (CC) (Furfaro et al., 2018).

The evolutionary relationships among STs, representing 3,456 nucleotides of the seven MLST genes, were inferred using the neighbor-joining (NJ) method. Evolutionary distances were calculated using the *p*-distance method, which considers the number of different nucleotides out of the total, and an NJ tree with 1,000 bootstrap replications was constructed using MEGA5 software (Tamura et al., 2011).

The discriminatory power of the different methods, alone or in association, was evaluated using the Simpson’s index of diversity (Mokrousov, 2017), using a free online tool^2^.

### Antimicrobial Susceptibility Testing

The susceptibility of the isolates was analyzed using the disc diffusion method according to the Clinical and Laboratory Standard Institute (CLSI) guidelines (Clinical and Laboratory Standard Institute (CLSI), 2018b). The zone diameter interpretative standards of CLSI-M100 Ed29 and CLSI-VET08 Ed4 (Clinical and Laboratory Standard Institute (CLSI), 2018a, Clinical and Laboratory Standard Institute (CLSI), 2019) were used for interpretation.

The antimicrobial tests were those included in the laboratory panel for mastitis Gram-positive pathogens of IZSLER. The molecules used are representative antimicrobials that can predict susceptibility to other members of the same class, according to the guidelines of the Italian Reference Centre for Antimicrobial Resistance^3^.

The following antimicrobials were tested: amoxicillin-clavulanic acid (20–10 μg), ampicillin (10 μg), cephalothin (30 μg), ceftiofur (30 μg), erythromycin (15 μg), kanamycin (30 μg), penicillin G (10 IU, International Units), pirlimycin (2 μg), rifampicin (5 μg), sulfisoxazole (300 μg), tetracycline (30 μg), and trimethoprim-sulfamethoxazole (1.25–23.75 μg).

## Results

### MCT and LT

#### All Selected Isolates Were Confirmed to Be *S. agalactiae* Using Species-Specific PCR

The results of MCT on the selected cattle and human isolates are reported in Supplementary Tables 1, 2, respectively. The MCT results summarized in Table 2 show that serotypes Ia, Ib, II, III, IV, and V were common to human and bovine isolates, while there were no isolates with serotypes VI, VII, and VIII in either cattle or humans.

**TABLE 2:**
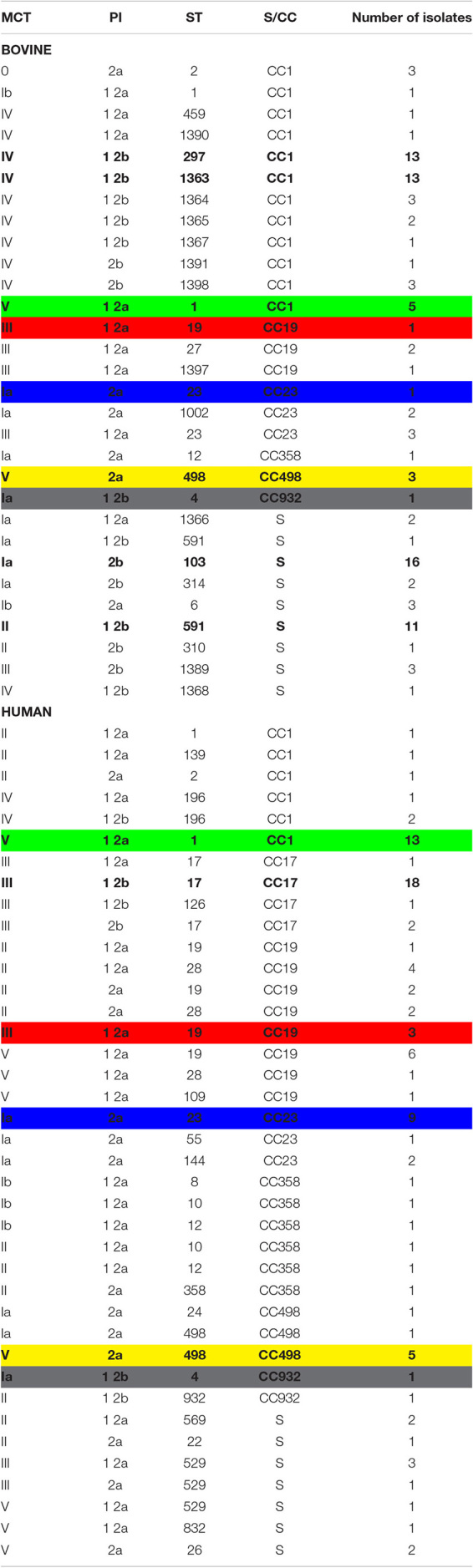
Results of the molecular capsular typing, pilus island typing, and multi-locus sequence typing of 201 *Streptococcus agalactiae* isolates.

*The most frequently combined genotypes are in bold. Common combined genotypes between bovines and humans are put in evidence by the same color.*

In bovines, serotype IV was predominant in 39 out of 103 (37.9%) isolates, followed by Ia in 26 out of 103 (25.2%), while three isolates (2.9%) did not return any PCR results (Supplementary Table 1). In humans, the predominant serotype was V in 31 out of 100 cases (31.0%) (Supplementary Table 2).

The ability to ferment lactose was common in cattle (98.1%) and rare (9.0%) in human isolates.

### PI Typing

The PI-1/2b profile was the most common type among bovine isolates (44.7%), while the PI-1/2a profile was the most common among human isolates (46.0%) (Table 2 and Supplementary Tables 1, 2).

### MLST

#### A Total of 45 Different STs Were Obtained From MLST Analysis of All 203 Isolates

Specifically, 27 different STs were obtained among the 103 bovine isolates, 11 of which were new and deposited in PubMLST (ST1363-ST1368, ST1389-ST1391, and ST1397-ST1398). The most frequent profiles were ST103 (16/103, 15.5%), ST297 (13/103, 12.6%), ST1363 (13/103, 12.6%), and ST591 (12/103, 11.7%) (Supplementary Table 1). In five bovine isolates, indicated with an asterisk in Supplementary Table 1, PCR amplification of the *glcK* gene produced a 3.0-kb band instead of the expected 0.5-kb band. This increase in the fragment size was found to be due to a mobile genetic element which was inserted at an identical point in the *glcK* gene in each of these five isolates. As MLST analysis creates concatenated sequences, the nucleotide sequence of the mobile element was ignored and removed, leaving the intact *glcK* sequence corresponding to allele 1 for all isolates.

Among the 100 human isolates, 25 different STs already described were obtained (Jolley et al., 2018), and the most frequent STs were ST17 (21/100, 21.0%), ST1 (14/100, 14.0%), ST19 (10/100, 10.0%), and ST23 (9/100, 9.0%) (Supplementary Table 2). The most frequent STs were isolates from both symptomatic and asymptomatic human cases.

The common STs in both groups were ST1, ST2, ST4, ST12, ST19, ST23, and ST498 (Table 2 and Supplementary Tables 1, 2).

**FIGURE 2:**
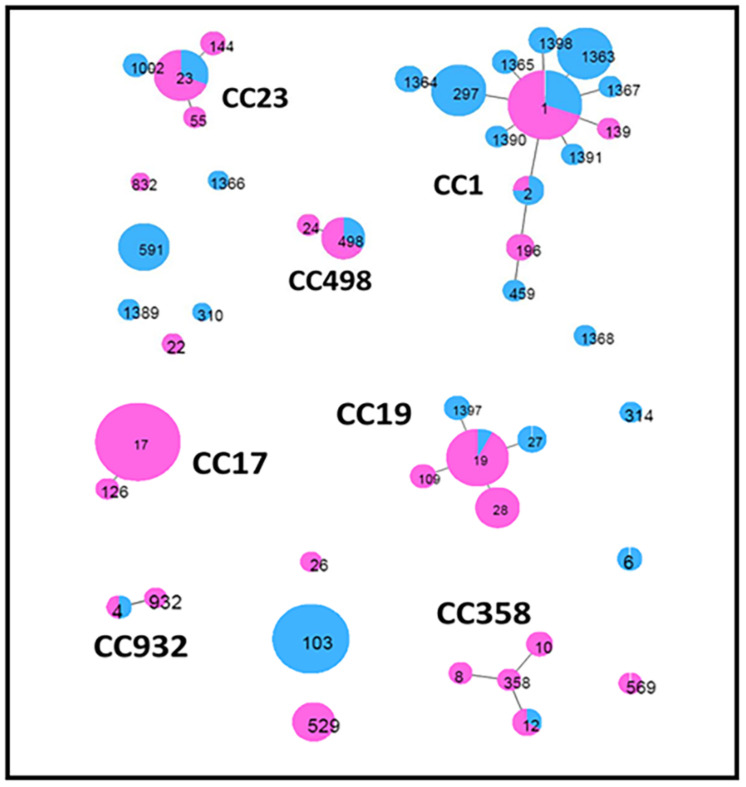
Minimum spanning tree obtained by global eBurst analysis using PHYLOVIZ software. This shows the distribution of host species across clusters of *Streptococcus agalactiae* sequence types (STs) obtained in the present study. Each circle represents an ST. The size of the circle and its colored segments are proportional to the number and origin of isolates, where pink refers to humans and blue refers to bovines, respectively. Clusters, including single- and double-locus variants, are indicated by the corresponding clonal complex (CC).

Global eBURST analysis (Figure 2) showed that 13 STs were singletons, not belonging to any CC, eight of which were from cattle and five from humans (Table 2). The remaining 32 STs, with single- and double-locus variants, were grouped into seven CCs: CC17 was exclusively found in human isolates, while the other six (CC1, CC19, CC23, CC358, CC498, and CC932) were common to both groups.

**FIGURE 3:**
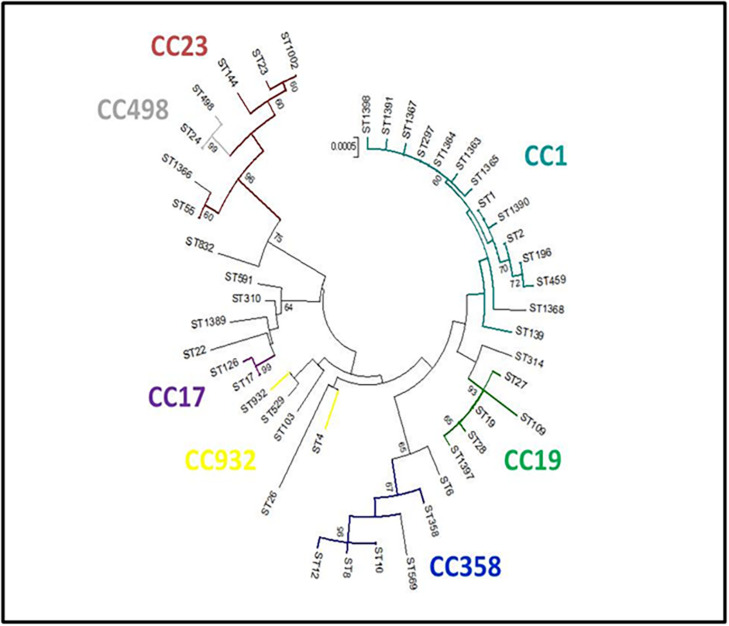
Neighbor-joining (NJ) tree. Genetic relationships among the 45 *S. agalactiae* sequence types (STs) obtained in the present study were inferred with MEGA5 software [14]. Evolutionary distances were calculated using the *p*-distance method, which considers the number of different nucleotides out of the total (3,456). A NJ tree with 1,000 bootstrap replications was constructed. The clonal complexes (CCs) obtained by the global eBURST analysis are reported.

The evolutionary relationships of the 45 ST profiles are shown in an NJ phylogenetic tree (Figure 3), where we have underlined how the different genotypes are related to each other. This may explain, for example, the presence in the same herd (PC06) of isolates with different sequence types (ST19 and ST27), which, although different, are evolutionarily related and belong to the same CC (CC19).

### Combination of Typing Methods

Considering the combination of the three typing methods (MCT-PI-MLST), it was possible to obtain 64 combined profiles for 201 out of 203 isolates; two isolates were excluded because their MLST profiles were incomplete (Supplementary Tables 1, 2).

Simpson’s index of diversity was 0.962 (95% CI: 0.953–0.971). The most frequent combined profiles are shown in bold in Table 2, where the five allelic profiles common to bovines and humans (V-1 2a-ST1, III-1 2a-ST19, Ia-2a-ST23, V-2a-ST498, and Ia-12b-ST4) are marked in the same colors.

By analyzing the MCT results, we found the presence of sympatric field isolates belonging to different serotypes in two herds: serotypes Ia and IV in herd PR2, and serotypes Ib and V in herd PR15. This observation was confirmed by the MLST analysis. In herd PR2, we observed the presence of isolates ST103 and ST297, which were associated with serotypes Ia and IV, respectively, whereas in herd PR15, ST1, and ST498 were associated with serotypes Ib and V, respectively.

### Antimicrobial Susceptibility Testing

#### Table 3 Shows the Percentages of Isolates Resistant to the Main Classes of Antibiotics

**TABLE 3:** Antimicrobial susceptibility testing.

	Bovine isolates not susceptible (intermediated or resistant) *N* = 103	Human isolates not susceptible (intermediated or resistant) *N* = 100
Amoxicillin-clavulanic acid	0	0
Ampicillin	0	0
Cephalothin	0	0
Ceftiofur	0	0
Erythromycin	11 (10.7%)	15 (15%)
Kanamycin	103 (100%)	100 (100%)
Penicillin G	0	0
Pirlimycin	9 (8.7%)	12 (12%)
Rifampicin	0	0
Sulfisoxazole	23 (22.3%)	44 (44%)
Tetracycline	32 (31.1%)	36 (36%)
Trimethoprim – sulfamethoxazole	1 (1.0%)	0

*The percentages of isolates by origin (bovine and human) resistant to representative molecules of the main classes of antibiotics.*

All field isolates were sensitive to beta-lactam antibiotics (amoxicillin-clavulanic acid, ampicillin, cephalothin, ceftiofur, and penicillin G) and rifampicin.

All field isolates were resistant to aminoglycosides (representative molecule: kanamycin) because of the intrinsic resistance of *Streptococcus* to this category of molecules.

Different proportions of isolates resistant to erythromycin, pirlimycin, sulfisoxazole, and tetracycline were observed in both groups. In particular, resistance to sulfisoxazole and tetracycline was the most widespread in both groups.

The resistance of human and bovine GBSs to these antibiotics was associated with many different serotypes and STs, as reported in Table 4. We observed two bovine III-ST23 and eight human Ia-ST23 isolates that were resistant to sulfisoxazole and/or tetracycline. In addition, many human and bovine 1 2a-ST1 and 2a-ST498 isolates, in combination with serotype V, are often resistant to one or more of the following antibiotics: erythromycin, pirlimycin, sulfisoxazole, and tetracycline.

**TABLE 4:** Molecular capsular typing and sequence types of the bovine and human resistant isolates.

	Bovine isolates		Human isolates	
	MCT	ST	MCT	ST
Erythromycin	Ia	12, 1,366		
	Ib	1	Ib	10
	II	591 (2)	II	19, 22, 28
	V	1, 498 (3)	V	1 (7), 19, 109, 498, NT
	NT	2		
Kanamycin		All strains		All strains
Pirlimycin	Ib	1	Ib	10
	III	1,397	II	19, 22
	IV	459, 1,363		
	V	1 (2), 498	V	1 (7), 19, NT
	NT	2 (2)		
Sulfisoxazole	Ia	4, 103 (4), 591, 1002, 1366	Ia	4, 23 (8), 55, 144
			Ib	8, 12
	II	591 (7)	II	10, 12, 19 (2), 22, 28 (4), 932
	III	23 (2)	III	17 (10), 19 (2)
	IV	297 (3), 1363, 1365	IV	196
	V	1	V	1 (2), 19 (2), 26, 498, 832, NT
Tetracycline	Ia	12, 23, 103 (11), 314 (2), 1002 (2)	Ia	23 (4), 24, 498
	Ib	1, 6 (3)	Ib	12
			II	1, 12, 19 (2), 22, 28 (2), 569, 932
	III	27, NT	III	17 (5), 529 (2)
	IV	297, 459, 1363, 1398,	IV	196 (2)
	V	498 (3)	V	1 (4), 19, 26 (2), 498, 529, 832, NT
	NT	2 (2)		
Trimethoprim – sulfamethoxazole	V	1		

*MCT, molecular capsular typing; ST, sequence type.*

Human ST19 isolates associated with serotype II or V showed multi-resistance, while all bovine ST19 isolates were susceptible to all antibiotics.

Notably, we observed one human and one bovine isolate, both from the same province, with the same combined Ia-1 2b-ST4 profile, showing resistance to sulfisoxazole.

Finally, among the most common human and bovine isolates (those showing profiles III-ST17 and Ia-ST103, respectively), we often observed resistance to sulfisoxazole and/or tetracycline.

## Discussion

To investigate the zoonotic potential of *S. agalactiae* in Italy, a collection of 203 sympatric *S. agalactiae* isolates from both humans and cattle, isolated in the same time frame (2018) and in the same geographic area (Emilia Romagna region, Northern Italy), were characterized.

Previously, Gherardi et al. (2007) and De Francesco et al. (2012) provided insight into the correlation among clonal types, serotypes, surface proteins, and antibiotic resistance of many Italian human isolates, highlighting clonal spread of the Italian GBSs. On the contrary, no studies on cattle isolates from Italian herds are so far available in the international literature.

Recently Lyhs et al. (2016) analyzed many sympatric isolates from humans and cattle in Finland and Sweden and highlighted how the same GBS subtypes were present in both species.

With regard to serotypes, serotype V was the most widespread in both species in Finland and Sweden (Lyhs et al., 2016). In Spain (Rojo-Bezares et al., 2016), serotype III (associated with serious infections in humans) was predominant in humans, while in Iran (Emaneini et al., 2016), serotype III was predominant in both humans and cattle, followed by serotype V in humans. In Algeria and France, serotype V was the most prevalent among human isolates (Bergal et al., 2015). In the United States, there was an increase in the prevalence of human isolates with serotype IV from 1990–2010 (Diedrick et al., 2010); moreover, they observed a higher representation of serotype Ia compared to the rest of the world (Ippolito et al., 2010). In our study, serotypes IV and Ia were the most prevalent in cattle, whereas serotype V was prevalent in humans, confirming the widespread diffusion of this serotype in humans (Gherardi et al., 2007; De Francesco et al., 2012; Bergal et al., 2015; Emaneini et al., 2016; Lyhs et al., 2016; Furfaro et al., 2018). We found three cattle isolates that could not be typed by MCT, as previously observed (Diedrick et al., 2010). This could be related to mutations in the alignment region of primers, the presence of non-specific capsular types, or absence of the capsule, as hypothesized by Carvalho-Castro et al. (2017). In contrast, the presence of mobile genetic elements in five bovine isolates recovered in the present study seems to be related to a particular bovine GBS lineage (Bisharat et al., 2004; Martins et al., 2010).

Notably, the ability to ferment lactose by almost all the cattle isolates may be due to a host adaptation mechanism in the cattle udder environment, as suggested by Lyhs et al. (2016).

PI typing was performed to verify the assignment of the field isolates to the possible profiles (PI-1, PI-1/2a, PI-1/2b, PI 2a, and PI 2b). Given the role of pili in GBS colonization and disease progression, the type of pilus affects GBS colonization and invasion of host cells (Richards et al., 2011). In this regard, we observed that the PI-1/2b profile and PI-1/2a were the most common types among bovine and human isolates, respectively, in line with previous reports (Springman et al., 2014; Lyhs et al., 2016; Shabayek and Spellerberg, 2018).

Out of the 45 different STs obtained by MLST analysis, 32 STs (corresponding to 74,6% of total) were grouped into seven CCs, with large differences within groups of clones. The most prevalent CCs were CC1, CC19, CC17, and CC23, as previously observed (Lyhs et al., 2016). Interestingly, ST1 (CC1), ST2 (CC1), ST4 (CC932), ST12 (CC358), ST19 (CC19), ST23 (CC23), and ST498 (CC498) profiles were common in cattle and humans. ST498, which has so far been isolated only in humans, was found in three cattle isolates in our study, suggesting a possible transfer between the two species.

Among human isolates, four STs (ST17, ST1, ST19, and ST23), corresponding to 56,6% of all STs, were the most widespread, as previously reported (Bisharat et al., 2004; Gherardi et al., 2007; De Francesco et al., 2012; Botelho et al., 2018; Furfaro et al., 2018). Notably, the main STs included in CC1 (ST1) and CC19 (ST19), in combination with different serotypes, have been associated with healthy carriers of Guillain-Barré syndrome and are responsible for the majority of GBS infections (Poyart et al., 2007; Bergal et al., 2015). The data obtained confirm that ST17, which is associated with capsular type III, has a known host-specific human profile, is associated with invasive neonatal diseases (Martins et al., 2010; Bergal et al., 2015; Lyhs et al., 2016; Lannes-Costa et al., 2020), and is one of the most frequent human STs, mainly in asymptomatic pregnant women. ST196 (serotype IV) was found in only three human isolates: one from a biopsy and two from vaginal swabs. These findings are relevant because GBSs with ST196 profiles are currently recognized as emerging pathogens for humans and have also been reported in cattle, which may therefore represent a potential reservoir (Lyhs et al., 2016).

Among bovine isolates, ST103 (S), ST297 (CC1), ST1363 (CC1), and ST591 (S) were the most prevalent, accounting for 52.4% of all bovine isolates. ST103 (serotype Ia), commonly reported among bovine isolates (Ippolito et al., 2010; Zadoks et al., 2011; Jørgensen et al., 2016; Lyhs et al., 2016; Botelho et al., 2018), was confirmed to be the most common type in cattle. Moreover, in some countries, ST103 has been circulating for a longer time (Zadoks et al., 2011) than elsewhere. In particular, in Brazil, ST103 was recovered from bovine and human isolates in the 1980s (Oliveira et al., 2006), where it has been reported to colonize the gastrointestinal tract of cattle, survive in the environment, and adapt well to the fecal-oral transmission pathway. These characteristics could be a critical point for *S. agalactiae* eradication in infected herds, unless appropriate environmental sanitation measures are put in place (Jørgensen et al., 2016). In Denmark, ST103 has only recently been detected as a consequence of the re-emergence of GBS as a significant cause of bovine mastitis (Zadoks et al., 2011). In the present study, ST103, even if it is the most frequent ST among bovine isolates, is not included in any CC and was detected only in this species, possibly because it was only recently introduced in cattle in this area.

In addition, we observed the circulation of isolates with different serotypes, PIs, and STs within the same farm. This observation highlights the possibility of co-infection caused by multiple genetically distinct isolates within the same herd. This may be attributed to the lack of biosecurity programs, or because of the introduction of cows with *S. agalactiae* infection or to different reservoirs of this pathogen other than infected mammary glands (Jørgensen et al., 2016; Tomazi et al., 2018).

Notably, previous studies (Lyhs et al., 2016; Botelho et al., 2018) did not report a Simpson’s index of diversity, while in the present study, the combination of MCT, PI typing, and MLST resulted in a Simpson’s index of diversity of 0.962 (95% CI: 0.953–0.971). This underlines the possibility of recovery of isolates with the same allelic profile in both cattle and humans, reinforcing the possibility of transmission between these two groups.

This hypothesis is supported by the observation that isolates from both groups not only share the same genotypes, but also the same antibiotic resistance profiles.

In addition, we noticed that human isolates always showed a higher rate of resistance to sulphamidic and tetracycline antibiotics than cattle strains, independent of their allelic profiles. In accordance with other studies (Gherardi et al., 2007; De Francesco et al., 2012; Bergal et al., 2015), most resistant isolates in the present study belonged to serotype V and CC-1, CC-498, and CC-19 clonal groups. Moreover, the resistance of *S. agalactiae* to erythromycin seems to be lower in cattle than in human isolates, with some differences among clusters, as previously reported (Lyhs et al., 2016; Tomazi et al., 2018). The resistance to erythromycin (10.7% and 15.0% in bovine and human isolates, respectively) seems to agree with the 15% of resistant isolates reported in a previous Italian study in humans (De Francesco et al., 2012), but lower than 29% and 40% recently observed by Tomazi et al. (2018) in bovine isolates from Brazil and by Bergal et al. (2015) in isolates recovered from clinical human cases in Algeria and France, respectively.

In conclusion, by analyzing sympatric GBS isolates, we found common isolates of *S. agalactiae* circulating both in cattle and human groups in the Emilia Romagna region, for a total of 20,9% (42/201) of common isolates. These results are supported by the high discriminatory power (>96.2%) of the combined typing methods used in this study.

## Data Availability Statement

The datasets presented in this study can be found in online repositories. The names of the repository/repositories and accession number(s) can be found in the article/Supplementary Material.

## Author Contributions

NA, GM, and AS: conceptualization. EC, CG, MC, and RS: data input and cleaning. EC and AM: data collection and analysis. NA, GM, and EC: funding acquisition. SR, AM, FB, CG, MC, RS, PB, and AP: investigation. NA, MR, EC, and AS: supervision. EC and NA: writing the original draft. EC, MR, NA, and AS: writing, review, and editing. All authors contributed to the article and approved the submitted version.

## Conflict of Interest

The authors declare that the research was conducted in the absence of any commercial or financial relationships that could be construed as a potential conflict of interest.

## Footnotes

1 http://pubmlst.org/sagalactiae/

2 http://www.comparingpartitions.info/?link=Tool

3 www.izslt.it./crab/linee-guida-per-linterpretazione-delle-prove-di-sensibilita-ai-chemioantibiotici-*in vitro*-per-un-utilizzo-nella-terapia-clinica/
